# Comprehensive deciphering the alternative splicing patterns involved in leaf morphogenesis of *Liriodendron chinense*

**DOI:** 10.1186/s12870-024-04915-x

**Published:** 2024-04-06

**Authors:** Yaxian Zong, Fengchao Zhang, Hainan Wu, Hui Xia, Junpeng Wu, Zhonghua Tu, Lichun Yang, Huogen Li

**Affiliations:** State Key Laboratory of Tree Genetics and Breeding, Co-Innovation Center for Sustainable Forestry in Southern China, Nanjing Forestry University, Nanjing, China

**Keywords:** *Liriodendron chinense*, Transcriptome, Alternative splicing, Leaf morphogenesis, *LcAIL5*

## Abstract

Alternative splicing (AS), a pivotal post-transcriptional regulatory mechanism, profoundly amplifies diversity and complexity of transcriptome and proteome. *Liriodendron chinense* (Hemsl.) Sarg., an excellent ornamental tree species renowned for its distinctive leaf shape, which resembles the mandarin jacket. Despite the documented potential genes related to leaf development of *L. chinense*, the underlying post-transcriptional regulatory mechanisms remain veiled. Here, we conducted a comprehensive analysis of the transcriptome to clarify the genome-wide landscape of the AS pattern and the spectrum of spliced isoforms during leaf developmental stages in *L. chinense*. Our investigation unveiled 50,259 AS events, involving 10,685 genes (32.9%), with intron retention as the most prevalent events. Notably, the initial stage of leaf development witnessed the detection of 804 differentially AS events affiliated with 548 genes. Although both differentially alternative splicing genes (DASGs) and differentially expressed genes (DEGs) were enriched into morphogenetic related pathways during the transition from fishhook (P2) to lobed (P7) leaves, there was only a modest degree of overlap between DASGs and DEGs. Furthermore, we conducted a comprehensively AS analysis on homologous genes involved in leaf morphogenesis, and most of which are subject to post-transcriptional regulation of AS. Among them, the *AINTEGUMENTA-LIKE* transcript factor *LcAIL5* was characterization in detailed, which experiences skipping exon (SE), and two transcripts displayed disparate expression patterns across multiple stages. Overall, these findings yield a comprehensive understanding of leaf development regulation via AS, offering a novel perspective for further deciphering the mechanism of plant leaf morphogenesis.

**Supplementary Information:**

The online version contains supplementary material available at 10.1186/s12870-024-04915-x.

## Introduction

In eukaryotes, precursor RNA (pre-mRNA) splicing, which removed the introns from the nascent mRNAs and ligated the exons in the right order, is an essential step in translatable mRNA synthesis and gene expression [1, 2]. Besides constitutive splicing in per-mRNAs, spliceosome flexibly selected splice site to generate structurally variable transcripts from individual pre-mRNAs, known as alternative splicing (AS) [3]. As an important post-transcriptional regulation mechanism, AS can greatly enhance genome coding capacity, transcriptome plasticity and proteome diversity [4]. Genome-wide analysis of AS have been shown that extensively influence diverse biological processes in plants. These include responses to various environmental stresses and physiological signals, along with the dynamic regulation of plant development such as fruit development and early plant morphogenesis [5–10]. However, some alternative transcripts might not necessarily perform biological functions. AS often impacts mRNA stability by introducing premature termination codons (PTCs). Transcripts harboring PTCs are targeted by the nonsense-mediated decay (NMD) pathway and rapidly degraded to maintain mRNA stability [11, 12].

The occurrence of alternative splicing is contingent upon the spliceosome, a complex comprising five small nuclear ribonucleoprotein (snRNPs) particles (U1, U2, U4, U5, and U6) and an array of non-snRNP proteins, including the U2 auxiliary factor (U2AF) [11, 13]. These RNA-protein complexes effectively recognize the 5’ and 3’ splicing sites and define exons and introns in a precise and ordered assembly [14]. Furthermore, trans-acting splice factors including serine/arginine-rich (SR) proteins and heterogeneous nuclear ribonucleoproteins (hnRNPs) also regulate the occurrence of AS through binding to *cis*-regulatory elements within exons (ESRs) and introns (ISRs), respectively [15, 16]. Categorically based on splice site selection, five distinct alternatively spliced subtypes have been identified in plants: retained introns (RI), skipped exons (SE), alternative 5’ splice sites (A5SS), alternative 3’ splice sites (A3SS), and mutually exclusive exons (MEX) [17]. Nearly 95% of human genes experience alternative splicing, with SE standing out as the primarily form [18], whereas in plants, more than 60-85% of genes are regulated by AS, with RI being the most prevalent form [19–21]. Moreover, the distribution of various AS types may exhibit significant variability between dicots and monocots [22]. While the fundamental splicing mechanism remains comparatively conserved, the intricacies of intron processing and the principles of alternative splicing appear to have diverged among plant species. Consequently, a more comprehensive investigation is imperative in the domain of plants to unveil the multifaceted and diversified mechanisms governing alternative splicing.

Leaf morphology can be diverse to facilitate photosynthesis and gas exchange in plants [23]. Plant leaf development is a complex biological process in which plant cells coordinate cell growth, cell division and cell differentiation at the three axis of proximal-distal, medial-lateral and abaxial-adaxial [24]. Previous studies have shown that the regulation of transcription factors is widely involved in plant leaf development. For instance, the Class I *KNOTTED1*‐like homeobox proteins (*KNOXI*) and the *ASYMMETRIC LEAVES1*(*AS1*) transcription factors antagonistically regulate the initiation of leaf primordium [23, 25]. Another noteworthy factor, *CUP-SHAPED COTYLEDON2* (*CUC2)* and *PIN‐FORMED1*(*PIN1*) feedback regulate the auxin maximum value at the serrated tip of the leaf margin to promote the intricate sculpting of leaf margin morphology [26]. Moreover, *AINTEGUMENTA* (*ANT*) and *GA20ox1* regulated leaf size by influencing the contents of auxin and gibberellin (GA), thus reflecting the interconnectedness of growth mechanisms [27, 28]. It is apparent that most studies delved into transcriptional regulation, with limited researches focusing on the impact of post-transcriptional mechanisms on leaf morphogenesis and development. Yet, AS is being progressively considered an important machinery for modulating plant development and response to various environment stresses. Examination of global AS patterns in wild strawberry (*Fragaria vesca* L.) throughout different fruit development stages reveals that shifts in AS modes could potentially contribute to rapid changes in gene expression during fruit set [7]. GS3 is regulated by AS to fine-tune grain size in rice (*Oryza sativa* L.) [29]. Furthermore, large-scale studies in plants have concentrated on dissecting AS patterns and dynamic expression changes within their isoforms across different tissues or developmental phases [30, 31]. In general, the expression level of the constitutive transcripts is significantly higher than variable isoforms, however, certain conditions can elicit shifts in this expression pattern, leading to different dominant isoforms [32]. These events have been defined as differential transcript usage (DTU), a phenomenon that holds promise for further offering insights into the occurrence of AS or for pinpointing potential candidates for functional analysis [33].

As an excellent ornamental tree, *Liriodendron chinense* (Hemsl.) Sarg. is widely used in garden cultivation thanks to its unique leaf shape, gorgeous flowers and straight trunk. The distinctive ‘mandarin jacket’ leaf shape of *Liriodendron* is widely recognized, rendering it a valuable subject for unraveling mechanisms of leaf morphogenesis. Insights into the dynamic leaf development of *L. chinense* at the transcriptional level have suggested the potential involvement of various transcription factors and genes related to phytohormone metabolism in the process [34]. On this basis, preliminary functional analyses have been conducted on core genes such as *LcKNOX*, *LcCUC2-like* [35, 36]. However, previous investigations have predominantly focused on the transcriptional level, leaving a gap in the comprehensive exploration of its post-transcriptional regulatory mechanism. While Tu et al have provided an overview of genome-wide alternative splicing events in *L. chinense* [37], no attention has been paid to the specific biological process of leaf morphogenesis. It is noteworthy that the wealth of genome resources and transcriptome data, derived from multiple samples, provide the possibility for a profound and tailored analysis of alternative splicing at the post-transcription level [34, 37, 38]. To uncover genome-wide AS events during leaf morphogenesis of *L. chinense*, we embarked on multiple available transcriptomes were analyzed in this study [34, 37]. Firstly, we conducted Iso-seq to produce an enhanced genome annotation file. Subsequently, we reanalyzed the RNA-seq data using this improved annotation to retrieve genome-wide AS events. Then, differential splicing events and genes between different developmental stages of *L. chinense* leaves were identified using RNA-seq data. Finally, we meticulously annotated and characterized the transcripts involved in leaf morphogenesis. Taken together, through secondary in-depth analysis of the available transcriptome data, we revealed the alternative splicing landscape throughout the leaf morphogenesis of *L. chinense* and characterized key candidate genes regulated by AS. Our study provides a new perspective for further exploring the mechanism of plant leaf morphogenesis.

## Materials and methods

### Data sources and plant materials

Published PacBio full-length transcripts as well as Illumina data of *L. Chinense* were analyzed to unveil alternative splicing events. Total RNA from seven *L. chinense* tissues (shoot apices, leaves, bracts, sepals, petals, stamens and pistils, 21 samples) were mixed for SMRT bell libraries construction, while all samples also were used respectively for Illumina sequencing (PRJNA559687) [37]. Besides, Illumina RNA-seq data from different young leaves stages include fishhook shaped stage (P2) and deeper lobe stage (P7) were used for differential analysis to find alternative splicing genes regulating lobed leaf development (SRR8101043, SRR8101042, SRR8101041 and SRR8101040) [34]. In addition, plant materials used in the experiment were collected from the forest farm affiliated to Nanjing Forestry University, Jiangsu Province, China (119°13′20″E, 32°7′8″N). Various tissues were removed from a mature *L. chinense* tree that was originally from Lushan, Jiangxi Province (116°0′E, 29°32′N) and young leaves in distinct developmental stages were removed from an adulted tree originated from Wuyi Mountain, Fujian Province (117°0′E, 27°N).

### Reads mapping and transcript assembly

**Fig. 1:**
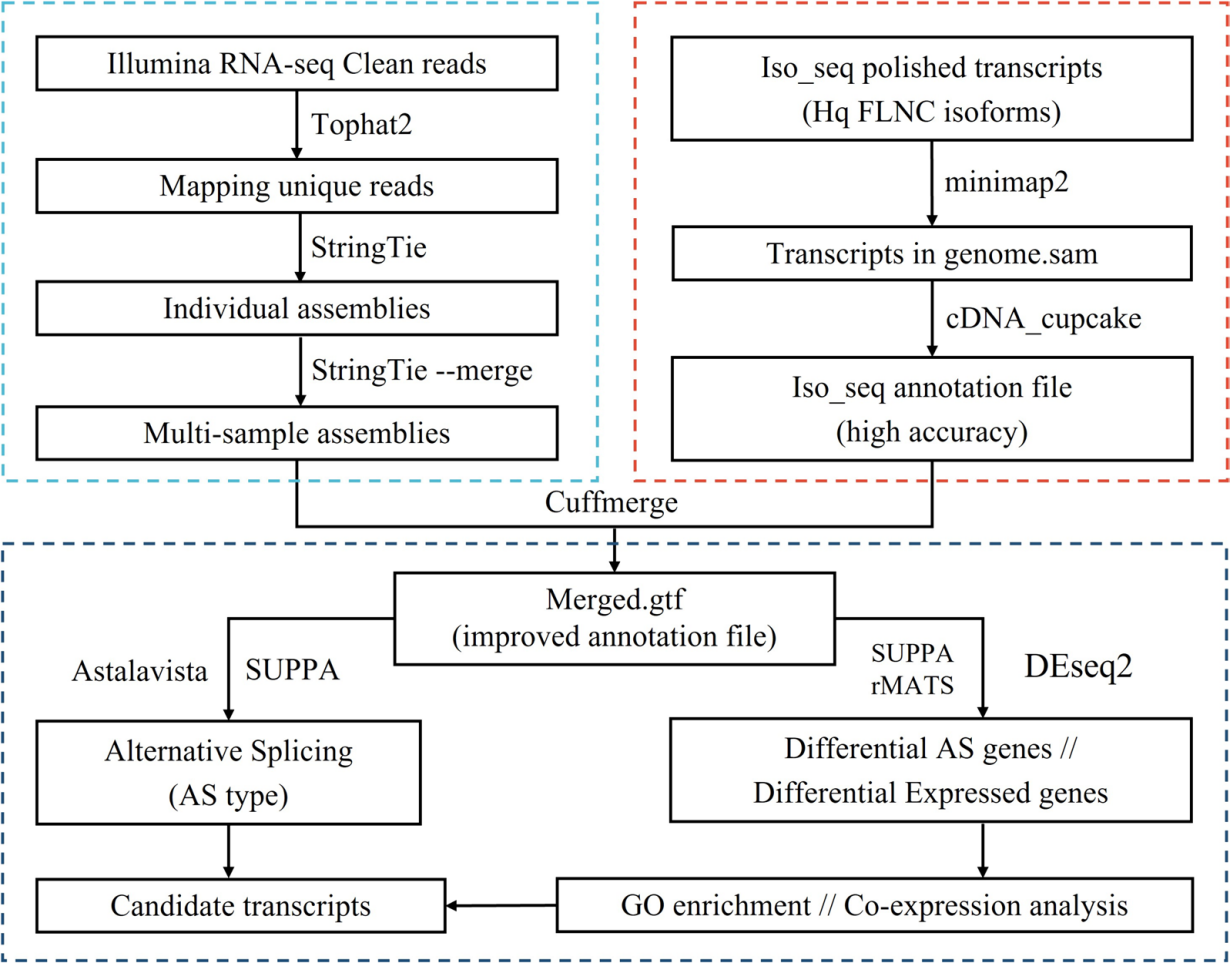
The workflow for acquiring the improved annotation file and AS analysis

An optimized pipeline was constructed for integrating transcriptome data resources (Fig. 1). Firstly, Illumina clean reads were mapped to the *L. Chinense* reference genome [38] using Tophat2 (v.2.1.1, http://ccb.jhu.edu/software/tophat/index.shtml) [39]. Based on alignment, the unique mapped reads were assembled by StringTie (v.2.2.1, https://ccb.jhu.edu/software/stringtie/) [40] with the default parameters. Then, the transcripts obtained from all samples were merged with StringTie --merge. Meanwhile, Minimap2 (v.2.24, https://github.com/lh3/minimap2) [41] was used to map high-quality full-length isoforms to the reference genome [42]. Mapped isoforms were further collapsed into non-redundant transcript groups using cDNA_Cupcake (v.29.0.0, https://github.com/Magdoll/cDNA_Cupcake).

To obtain an improved annotation file, transcripts from both short-read RNA-seq data and long-read Iso-seq data were integrated to consolidate transcript information and remove redundancy using cuffmerge in the Cufflinks suit (v.2.2.1, https://github.com/cole-trapnell-lab/cufflinks). Transcripts with class_code “=” (exact match of intron chain) or “j” (multi-exon with at least one junction match) were considered as isoforms of known genes [7]. Based on the above, the improved transcriptomes were used as reference for downstream analysis.

### Identification of leaf development stage differentially expressed genes (DEGs)

The fragments per kilobase per million mapped fragments (FPKM) values were calculated to estimate gene expression levels. The expression matrix of different stages was generated by cuffdiff in the Cufflinks suit (v.2.2.1, https://github.com/cole-trapnell-lab/cufflinks). DEseq2 (v.1.34.0, https://bioconductor.org/packages/DESeq2/) was used to calculate differential expression levels between two stages for leaf development [43]. Genes with *p-value* < 0.05 and |log2fold change| > 2 were further considered differentially expressed.

### Identification of AS events and discovery of differential alternative splicing genes (DASGs) between leaf development stages

To classify the AS events, SUPPA (v.2.3, https://github.com/comprna/SUPPA) [44] and AStalavista (v.4.0) [45] were employed using the annotation file assembled from Iso-seq and Illumina RNA-seq data. Five main types of AS events were extracted and counted, namely RI (structure: 0,1^2-), SE (structure: '0,1-2^), A3 (structure: 1-,2-), A5 (structure: 1^,2^), MXE (structure: 1-2^,3-4^). AS location was visualized based on the assembled transcripts using R package RIdeogram [46]. Base-composition bias was visualized using R package ggplot2 (v.3.4.1, https://ggplot2.tidyverse.org/) [47] and ggseqlogo [48].

Moreover, aligned data from shoot apices, leaves, P2 and P4 were further used to identify differentially alternative splicing events between leaf development stages by SUPPA with default parameters. The ‘psiPerEvent’ was performed to extract the proportion spliced-in (PSI) value of local AS events in each sample. Then, the events that are differentially spliced between a pair of conditions were calculated with ‘diffSplice’. Differential events were filtered with *P*-value < 0.05. The events examined include retained introns (RI), retained partial exons in 3’ splice site (A3SS) or 5’ splice site (A5SS), skipped exons (SE) and two mutually to exclusive exons (MXE). Finally, the sashimi plot was plotted based on the built-in rmats2sashimiplot function of Rmats.

### Gene Ontology (GO) enrichment analysis and visualization

The predicted coding region of transcripts were determined using TransDecoder (v.5.5.0, https://github.com/TransDecoder). Then, the protein from the longest ORF were functionally annotated using eggnog (v.5.0, http://eggnog5.embl.de/#/app-/home) [49]. Gene Ontology (GO) enrichment analysis was performed to explore the potential functions of DASGs and DEGs using the R package clusterProfiler (v.4.2.2, https://bioconductor.org/packages/release/bioc/html/cluster-Profiler.html) [50]. GO terms with *P-value* ≤ 0.01 were considered as significantly enriched categories. The R package ggplot2 (v.3.4.1, https://ggplot2.tidyverse.org/) was used for visualization.

### RT-qPCR of AS transcripts

Total RNA was extracted from samples with the RNA-prep pure kit (Tiangen, Beijing, China) according to the instructions. Then, cDNA was synthesized from 500ng total RNA using Evo M-MLV RT Premix AG11706 (Accurate Biotechnology (Hunan) Co.,Ltd) in a 10 μL reaction volume. Using Oligo 7.0 software, primers for RT- qPCR were designed adhering to the instructions.

### Phylogenetic analysis

Protein sequences of AIL5 isoforms from various plant species were obtained by conducting BLASTP search. These sequences were derived from NCBI database (https://www.ncbi.nlm.nih.gov/), FernBase (https://fernbase.org/) and multiple gymnosperms genomes [51–54]. Multiple sequences were aligned using Mafft (v 7.310) with default parameters [55]. Then, the unrooted phylogenetic tree was constructed using the maximum-likelihood (ML) method in IQ-TREE (v 1.6.12) [56]. The phylogenetic tree was visualized using iTOL (https://itol.embl.de/) online tool [57]. Gene structure was displayed using GSDS (v2.0, https://gsds.gao-lab.org/) [58].

## Results

### Overview of *Liriodendron Chinense* transcriptome assembly

To comprehensively investigate AS events in *Liriodendron Chinense*, we employed a dataset encompassing 25 RNA-seq samples. This dataset incorporated seven tissues (shoot apices, leaves, bracts, sepals, petals, stamens and pistils) as well as two leaf development stages (P2 and P7). Following alignment to the genome and the subsequent filtration of discordant and multiple alignments, an average of about 32.49 (82.31%) million unique mapped reads was utilized for subsequent transcriptional assembly (Supplementary Table S1). For each tissue, we independently constructed transcript assemblies according to a set of criteria. Then, the transcriptomes of these tissues were merged. Additionally, the annotation file was further improved by integrating the high-quality full-length isoforms and the reference genome. It should be noted that some transcripts were filtered out based on the “class code” and were not assembled within the reference annotation due to simple consideration for the main types of transcripts that match the reference genome. An updated annotation file was generated after filtering and removing redundancy as the reference to identify AS events (Supplementary File S1). From the annotation file, we identified a total of 90,376 transcripts associated with 32,445 genes. Notably, approximately 47% of these genes (15,306) featured multiple isoforms, collectively contributing 73,238 transcripts. Additionally, around 68.6% of transcripts (50,217) harbored more than one intron yet not exceeding ten introns.

### Alternative splicing landscape in *Liriodendron Chinense*

**Fig. 2:**
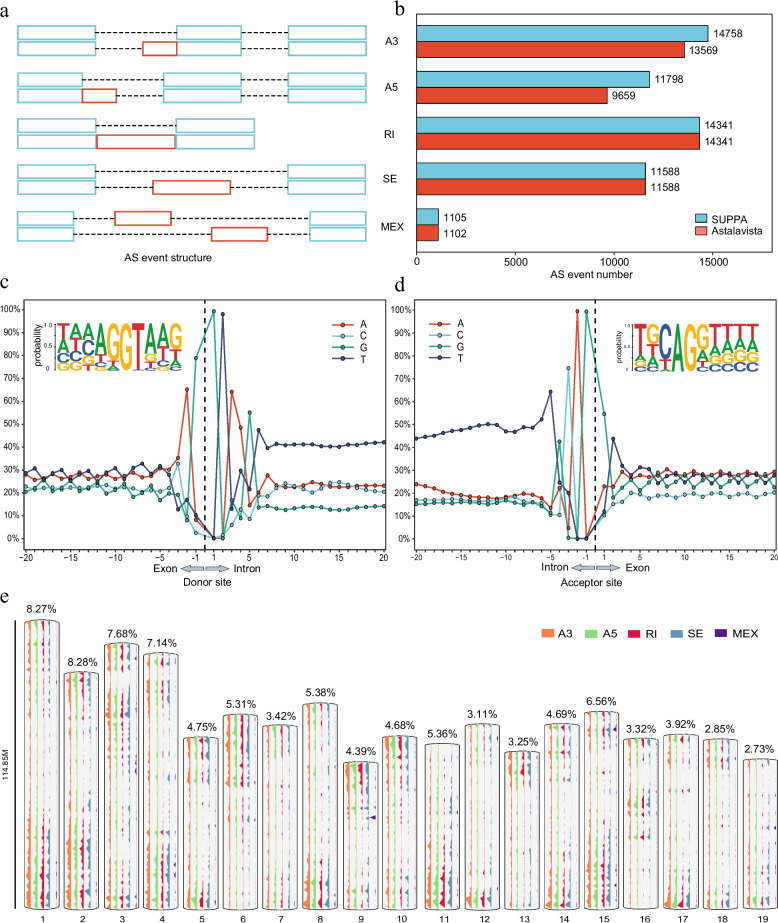
Genome-wide alternative splicing (AS) landscape of *L. Chinense*. **a** The five representative forms of AS, namely alternative 3’ splice sites (A3SS), alternative 5’ splice sites (A5SS), retained introns (RI), skipping exons (SE) and mutually exclusive exons (MEX). **b** The number of each AS type identified using different software. **c** and **d** Analysis of splicing site bias, including the donor site (**c**) and acceptor site (**d**). **e** Distribution of AS events on chromosomes. Different colors represent different types of AS

To uncover genome-wide alternative splicing AS events, we analyzed the annotation file through both SUPPA and AStalavista respectively and focused on the five major types of alternative splicing events (Fig. 2a). A total of 50,259 AS events were identified simultaneously involving 10,685 genes (32.9 %), including retained intron (IR, 14,341 events, 28.5 %), skipped exon (ES, 11,588 events, 23.1 %), alternative 3’ splice site (A3SS, 13,569 events, 27 %), alternative 5’ splice site (A5SS, 9,659 events, 19.2 %) and mutually exclusive exons (MEX, 1,102 events, 2.2 %) (Fig. 2b, Supplementary File S2). Among them, IR were the most abundant splicing events, followed by A3SS, while MEX were relatively less frequent. Notably, the occurrence rate of IR events in *L. chinense* is considerably lower than what is observed in other plants [59].

The process of AS depends on the effectively recognition of the 5’ and 3’ splicing sites by splicing factors. Therefore, we analyzed the nucleotide frequency of transcripts using .gtf file to unravel the distribution of nucleotide at splicing sites. The results showed that obvious nucleotide bias was found at the donor/acceptor site. Remarkably, the core sequence elements ‘GT’ (donor site) (Fig. 2c) and ‘AG’ (acceptor site) (Fig. 2d) were identified as highly conserved, constituting 97.45% of all splice sites. This finding is consistent with the previously reported “GT—AG” rule of remove introns [12]. Besides, the U2 type splice site “GC—AG” constituted 1.44% of all splice sites, and the U12 type splice site “AT—AC” accounted for 0.17% (Supplementary Table S2). Despite the low distribution frequency, these splicing site types without canonical borders are also relatively conserved during pre-mRNA splicing across species [60, 61].

Additionally, we drew chromosome distribution maps to visually represent the occurrence of five distinct types of alternative splicing events across the genome, with the exception of those splicing events distributed within contigs (Fig. 2e). The distribution of these AS events exhibited an uneven pattern across all 19 chromosomes, while following a consistent trend across the five splicing types, that is, more distribution at both ends of the chromosome and few in the middle. Intriguingly, the distribution of AS events did not exhibit a strict positive correlation with chromosome length. For instance, Chr 2 harbored the highest proportion of AS events at 8.28%, whereas Chr 19 displayed the lowest percentage at 2.73%. Notably, even the shortest Chr 9 contributed 4.39% to the overall tally of AS events.

### Characteristics of Alternative splicing events during leaf development

**Fig. 3:**
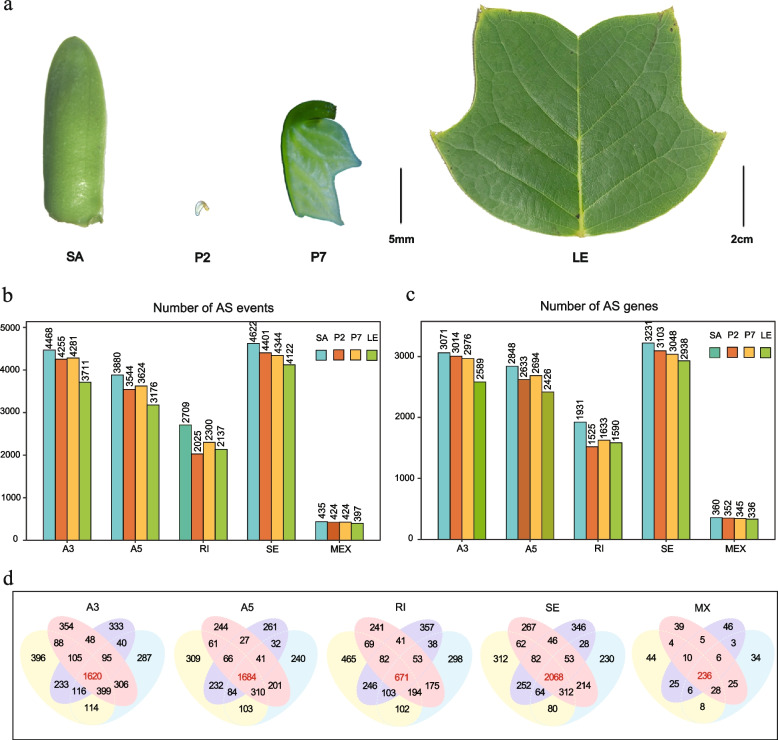
AS analysis at different stages of leaf development. **a** Four development stages of *L. Chinense* leaves, including shoot apices (SA), fishhook shaped stage (P2), deeper lobe stage (P7) and leaves (LE). **b** and **c** The number of each type of AS events (**b**) and genes (**c**) at different stages. **d** Venn diagrams of the same AS type at different development stages

Subsequently, we focused on the AS events during the leaf development, encompassing the five major types, A3SS, A5SS, RI, SE and MXE, aiming to explore the role of alternative splicing in leaf morphogenesis (Fig. 3a). Through the analysis of Illumina RNA-seq data spanning four stages of leaf development, a total of 16,114, 14,973, 14,649 and 13,543 alternative splicing events were identified in SA, P2, P7 and LE samples, respectively (Fig. 3b, c). Notably, from the early stage of leaf morphogenesis to mature leaves, this temporal analysis revealed a gradual reduction in the number of splicing events, suggesting a propensity for alternative splicing to primarily occur during the active growth stages.

Delving into the various splicing types, we observed that SE was the most abundant AS events, ranging from 28.7% to 30.4% across the four stages and involving the most AS genes (Fig. 3b, c). In contrast, RI events were less frequently detected, comprising only 13.8% to 16.8% of events and surpassing only MXE events in frequency. This distribution may be attributed to limitations in genome assembly quality and the short-read lengths of Illumina RNA-seq data. Furthermore, statistical analysis revealed that there were 1,620, 1,684, 671, 2,068, and 236 genes consistently undergoing A3SS, A5SS, RI, SE, and MXE events, respectively, across all four developmental stages (Fig. 3d), indicating the conservation of some AS events across different leaf development stages. However, the Venn diagram reveals that still numerous AS events are distinct at different developmental stages, indicating stage-specific regulation of AS (Fig. 3d).

### Analysis of DASGs and DEGs during leaf development and corresponding pathways enrichment

**Fig. 4:**
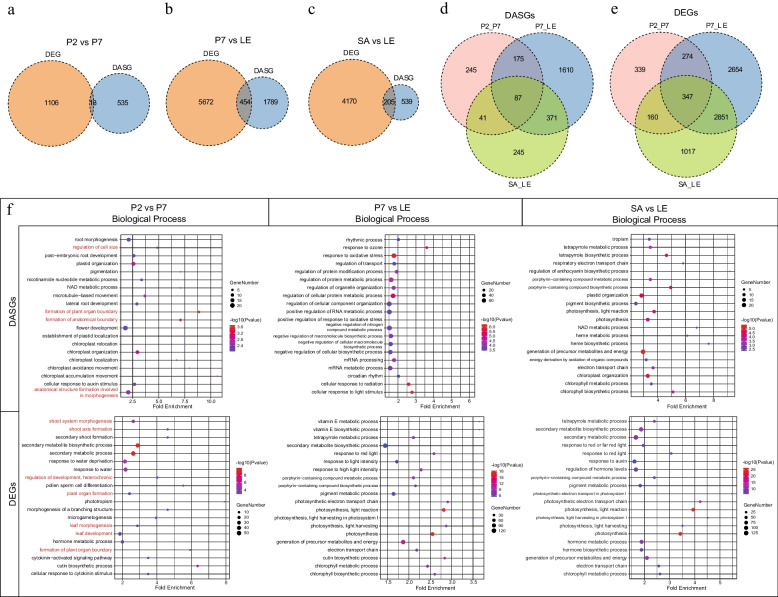
Differential alternative splicing genes (DASGs) and differentially expressed genes (DEGs) during leaf development, and corresponding pathways GO enrichment. **a**-**c** Overlaps between DASGs and DEGs during different developmental stages, including P2 vs P7 (**a**), P7 vs LE (**b**) and SA vs LE (**c**). **d** and **e** Venn diagrams of DASGs (**d**) and DEGs (**e**). **f** The top 20 most significantly enriched GO terms in biological process of DASGs and DEGs. The red font marks the pathways related to leaf morphogenesis

To gain a deeper understanding into the dynamic shifts in splicing that unfold during leaf development, we performed a comparative analysis of the relative expression of alternative splicing events across four developmental stages. Subsequently, the differentially alternative splicing genes (DASGs) and events were identified, sorted and integrated. (Supplementary Table S3). Correspondingly, the same comparative groups were applied to discern significantly differentially expressed genes (DEGs) (Supplementary Table S4). Based on statistical findings, a total of 804 DAS events, sourced from 548 gene loci, were delineated through the calculation of differences in mean PSI values between P2 and P7 stages (Fig. 4a). Notably, during the transition from young lobed (P7) to mature leaves (LE), 3,734 DAS events were detected with the highest frequency, from 2,243 gene loci (Fig. 4b). Besides, a comparative analysis between shoot apices (SA) and leaves (LE) led to the identification of 1,111 DAS events from 744 gene loci (Fig. 4c). However, only 87 genes were identified as shared DASGs in all three aforementioned comparisons (Fig. 4d). By contrast, 347 genes exhibited DEG commonality across the array of the comparisons (Fig. 4e). The formation of leaf organs is an orderly and complex process, in which different isoforms perform their functions at different stages.

As an important post-transcriptional regulatory mechanism, alternative splicing affects gene transcription and expression dynamics. To explore the potential relationship between alternative splicing and gene expression, we engaged in a comparative analysis of DASGs and DEGs. Intriguingly, only 13 genes (2.37%) with DAS events were affiliated with differentially expressed during the development of tender leaves (Fig. 4a). There are relatively more overlaps, 454 genes (20.24%), between differential splicing and differential expression during the transformation process of mature leaves (Fig. 4b). In the three comparisons, despite not exhibiting the largest overlap between differentially spliced and differentially expressed genes between leaf buds and leaves, they demonstrate the highest proportion (27.55%) in differentially spliced genes (Fig. 4c). The overall comparison revealed a limited number of genes that establish an intersection between DASGs and DEGs.

To investigate the influence of development-induced alternative splicing events on biological processes, we performed GO functional enrichment analysis on both DASGs and DEGs. During the transition from fishhook (P2) to lobed (P7) leaves, enrichment analysis revealed that genes with DAS events are primarily involved in developmental and morphogenetic pathways, such as regulation of cell size, formation of plant organ boundary and anatomical boundary (Fig. 4f). Notably, these biological processes coincided with the development stages of leaves. Comparatively, differentially expressed genes are also mainly enriched in development and morphogenetic pathways, especially leaf and shoot morphogenesis (Fig. 4f). Nonetheless, it is noteworthy that the cohort of DEGs captured by these pathways exhibits significant divergence from the subset of DASGs captured, with only one intersection *Lchi00862*, a homeobox transcription factor (Supplementary Table S5). Moreover, the analysis of differential genes during the transition to mature leaves showed that both DASGs and DEGs exhibited predominant involvement in responding to light stimulation and photosynthesis pathway (Fig. 4f). During this stage, leaves generate carbohydrates and ATPs for plant growth via photosynthesis and metabolic activities. Previous studies paid more attention to the involvement of DEGs in plant growth and development, while neglecting the regulation of DASGs. Through statistical and enrichment analysis, it was found that the regulation of post-transcriptional level was also closely related to plant growth and development, and is relatively independent from the transcriptional regulation.

### Features of DAS events in transcript isoforms during the transition from fishhook (P2) to lobed (P7) leaves

**Fig. 5:**
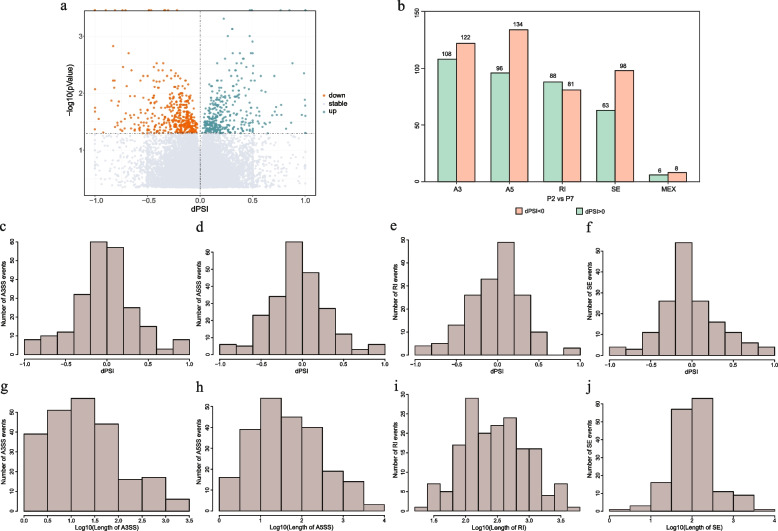
The features of DAS events between fishhook (P2) and lobed (P7) leaves. **a** Volcano map showing the differential PSI (dPSI) levels. **b** Statistical analysis of dPSI value of different AS type. **c**-**f** Distribution of the dPSI value of different AS type, including AS33 (**c**), A5SS (**d**), RI (**e**) and SE (**f**). (**g** to **j**) Distribution of the sizes of variable region of different AS type, including AS33 (**g**), A5SS (**h**), RI (i) and SE (**j**)

To specifically investigate the characteristics of DAS events during the initial stages of leaf development, we further focused on the comparison between P2 and P7. Proportion spliced-in (PSI) is utilized to quantify the relative abundances of the splicing events or transcript isoforms, while the difference of these relative abundances across conditions are denoted by differential splicing (dPSI). During the transition from fishhook (P2) to lobed (P7) leaves, dPSI tends to be lower than zero, encompassing 443 splicing events (55%, 443/804). This pattern indicated that developmental progression has resulted in an increased proportion of exclusion isoforms from differentially splicing genes (Fig. 5a).

Subsequently, we generated frequency distributions for inclusion or exclusion lengths and dPSI values, aiming to characterize different splicing types. The result showed that RI events predominantly exhibited dPSI values higher than zero, indicating a propensity for intron retention during development (Fig. 5b, e). Conversely, other splicing events, including SE, A3SS, A5SS and MEX, contain more events with dPSI less than zero, indicating that exons and 3’ and 5’ terminal sequences tend to be skipped during development (Fig. 5b-f). Furthermore, the length distribution of the alternative region is different for various splicing events. Skipped exons exhibited the broadest range, spanning from three to 8,367 nt, with a median length of 101 nt (Fig. 5g). In contrast, retained introns displayed a length distribution ranging from 25 to 5,817 nt, with a median length of 289 nt (Fig. 5h). The alternative splicing length of A3SS and A5SS were generally short, with a median length of 18 nt for A3SS and 38 nt for A5SS (Fig. 5i, j).

### Alternative splicing events analysis of homologous gene related to leaf morphogensis

**Fig. 6:**
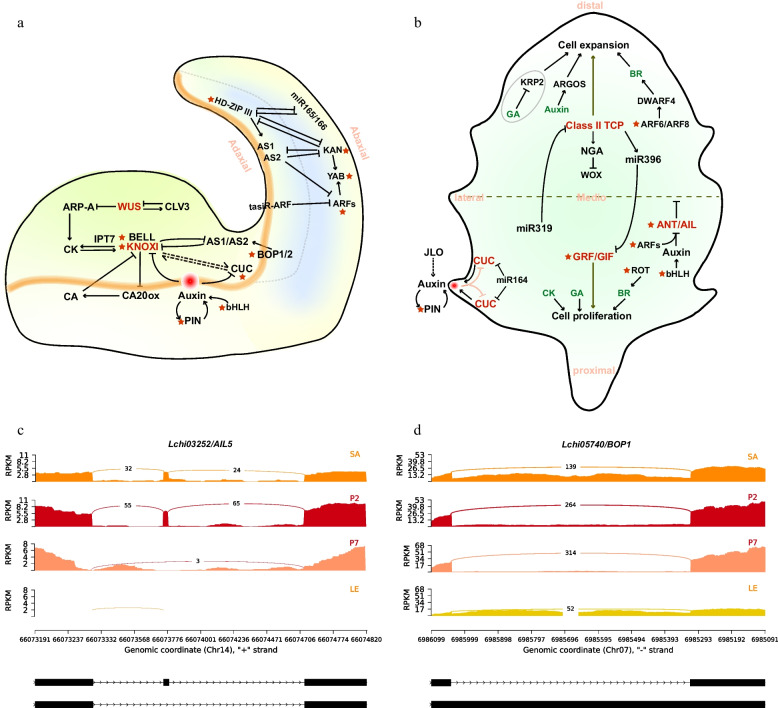
The genetic control of leaf morphogenesis based on previous reports. **a** Shoot apical meristems (SAM) maintenance and leaf primordia adaxial–abaxial polarity establishment. **b** Leaf growth along the proximal-distal and medio-lateral axis and leaf margin development. Red stars indicate homologous genes have undergone alternative splicing in *L. Chinense*, and details refer to Supplementary Table S6. **c** The sashimi plot of differentially spliced events of *Lchi03252*, which has been annotated as *AIL5* from APETALA2 transcription factor family. **d** The sashimi plot of differentially spliced events of *Lchi05740*, which has been annotated as *BOP1* acting on the adaxial side of leaf primordia

Based on previous research on leaf development, especially in the model plant *Arabidopsis thaliana* (L.) Heynh., we outlined a genetic mechanism network of leaf morphogenesis (Fig. 6a, b). Then, homologous genes in *L. Chinense* were obtained through protein sequence alignment, and some of these genes have been preliminarily validated for their functions in leaf development [35, 36]. Further survey revealed that some genes were regulated by alternative splicing post-transcriptional during leaf morphogenesis of *L. Chinense*, especially genes related to the leaf primordia initiation and the adaxial-abaxial polarity establishment (Supplementary Table S6). Two conservative mechanisms centered around KNOTTED1-like homeobox proteins (KNOXI) and PIN1 PIN-FORMED1 (PIN1) have been reported to be involved in leaf primordia, where the *LcKNAT6* as homologous gene of KNOXI contains RI and A5 events, while A3 and SE events were identified in *LcPIN1* gene. In addition, genes related to auxin homeostasis and transport, as well as plant growth regulation, such as *AINTEGUMENTA-LIKE/PLETHORA* (*AIL/PLT*), A*UXIN RESPONSE FACTORS* (*ARF*), *GROWTH-REGULATING FACTOR* (*GRF*) and *basic/HELIX–LOOP–HELIX TRANSCRIPTION FACTOR* (*bHLH*), are all involved in AS events. Regarding this phenomenon, it is speculated that during the initial stage of leaf development, external growth regulation and internal hormone homeostasis stimulate the expression of a substantial number of genes and the occurrence of AS events.

Subsequently, differential AS analysis was performed on these genes to investigate AS variation over development. The results showed that there were DAS events between the P2 and P7 stages for both *AIL* (*Lchi13837*, *Lchi03252*) and *ROTUNDIFOLIA (ROT*, *Lchi23114*), involving A5 and SE types (Supplementary Table S6). During the transition from P7 stage to mature leaves, DAS events were detected in *BLOCK OF CELL PROLIFERATION* (*BOP*, *Lchi05740*), *BEL1-LIKE HOMEODOMAIN* (*BELL*, *Lchi23624*) and *PIN1* (*Lchi16330*). In addition, the SE event of *ARF* (*Lchi18918*) was detected differently between shoot apices and mature leaves. Notably, we conducted a detailed unravelling analysis of two genes, *AIL5* (*Lchi03252*) and *BOP1* (*Lchi05740*). *AIL5* was significantly clustered into morphogenetic related pathways (Fig. 4f, Supplementary Table S5). This gene underwent an SE splicing event and had a significantly different between P2 and P7 stages (dPSI = -0.54, *p-value* = 0.005) (Fig. 6c). *BOP1*, a key gene that regulates the leaf’s proximal-distal patterning, produced an RI splicing event and significantly different between P7 and LE (dPSI = 0.43, *p-value* = 0.0025) (Fig. 6d). This spatiotemporal difference indicates that different isoforms could function at different development stages, these gene and its isoforms may be involved in leaf morphogenesis in ways that have not yet been explored.

### Conservation of alternative splicing in LcAIL5 orthologs across plants

**Fig. 7:**
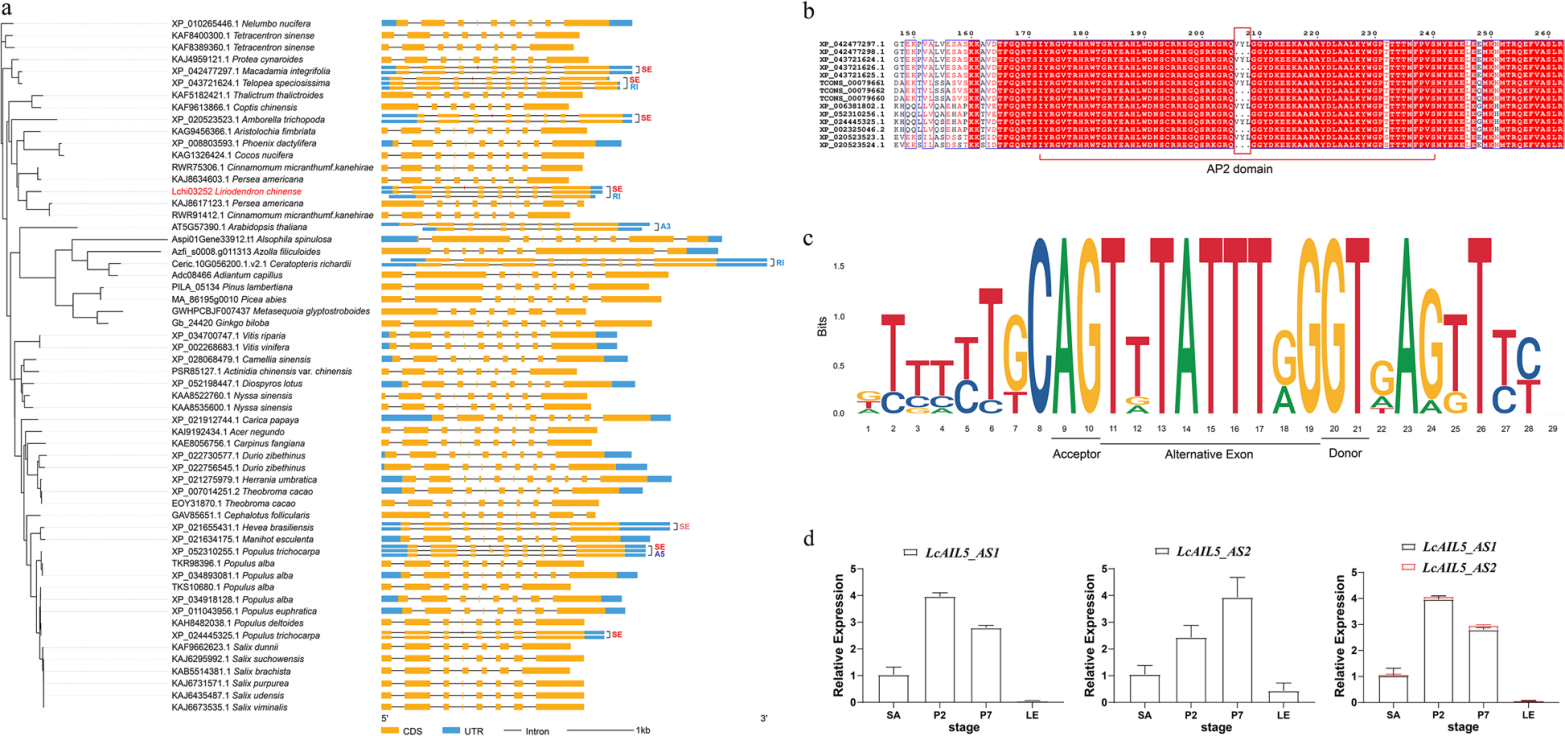
Phylogenetic and conservation analysis of *AIL5*. **a** Phylogenetic tree of *LcAIL5* homologous proteins. The unrooted phylogenetic tree was constructed using the maximum-likelihood (ML) method in IQ-TREE. Isoform structure was constructed using GSDS 2.0. Blue structure, UTR region; yellow structure, CDS; red box, variable exon. **b** Conserved protein domain alignment of isoforms with SE splicing event. **c** Base bias analysis of alternative exon and splice site of *AIL5* genes. **d** Relative expression of *LcAIL5_AS1* and *LcAIL5_AS2* in different developmental stages. The error bar shows Std. deviation

To further clarify the role of *AIL5* in plant growth and development through AS, we conducted an analysis of the AS patterns of *AIL5* orthologs across the plant kingdom. A total of protein sequences of 58 *AIL5* genes and gene structure information of 70 isoforms from 48 plant species were obtained by conducting BLASTP search, involving 40 angiosperms, four gymnosperms and four ferns. Subsequently, we constructed a phylogenetic tree to analyze their phylogenetic relationship using the protein sequences encoded by these isoforms (Fig. 7a). Based on current database information, we discovered nine *AIL5* genes with alternative splicing isoforms from eight species only. Since many sequences are retrieved from transcriptome data rather than a complete reference genome, the transcript information is not comprehensive and the actual number of isoforms may be far more than that. Nevertheless, an interesting phenomenon was found that seven out of the nine *AIL5* genes had experienced SE events. Except for *XP_021655431.1* gene, others all experienced the same forth exon skipped as *LcAIL5*. It is worth noting that the *AIL5* gene (*XP_020523523.1*) from *Amborella trichopoda*, a basal angiosperm closely related to *L. chinense*, also underwent the same alternative splicing event. This further indicates that the AS patterns of *AIL5* might exhibit evolutionarily conservation.

Through sequence alignment of proteins encoded by these genes and isoforms, it was found that the special exon that exhibited skipping encoded merely three amino acids, namely ‘VYL’. Notably, this motif is located in the first AP2 domain of AIL5 protein (Fig. 7b). Then, we performed a conservative analysis of the alternative exon and its 10 bp base near the two ends of the splice site (Fig. 7c). The result showed that these exon sequences were highly conserved except for the second and eighth bases. The acceptor and donor sites at the ends of the exon were ‘AG’ and ‘GT’ respectively, which conformed to the splicing rule [14]. Collectively, the same AS pattern exists in *LcAIL5* and its homologous genes, indicating a potentially evolutionarily conserved role in governing biological processes.

Subsequently, we designed primers for RT-qPCR to detect the expression patterns of *LcAIL5_AS1* (*TCONS_00079661*) and *LcAIL5_AS2* (*TCONS_00079662*) produced by SE (Fig. 7d). The result showed that the relative expression level of *LcAIL5_AS1* was higher in the early stage of leaf development (P2), and then gradually decreased. On the contrary, the relative expression level of *LcAIL5_AS2* was higher in late stages (P7). This is consistent with the transcriptome, suggesting that the two transcripts may be involved in leaf morphogenesis at different stages.

## Discussion

As an important post-transcriptional regulatory mechanism, alternative splicing (AS) is widely involved in various biological processes in plants [62, 63]. In addition, AS greatly enhances the coding capacity of the genome and enriches the diversity of the transcriptome and proteome [3]. Nowadays, high-throughput transcriptome sequencing and abundant genomic resources provide convenience for computational analysis of alternative splicing. AS has been widely explored in many plant species, including *Arabidopsis thaliana* [2, 64], rice (*Oryza sativa*) [20], maize (*Zea mays*) [16], cassava (*Manihot esculenta*) [6] and poplar (*Populus trichocarpa*) [8] and so on. Based on genome-wide integration analysis of RNA-seq data from multiple tissues and developmental stages, we detected more AS events in *L. Chinense* than previously reported [37]. In this study, approximately 32.9% (10,685/32,445) of genes were identified to undergo alternative splicing, involving a total of 50,259 AS events (Fig. 2b). This means that it might lead to encode more protein products that perform different functions [14]. A statistical classification of genome-wide AS events revealed that RI was the most abundant splicing event, aligning with the general characteristics of previous plant studies [65].

The process of alternative splicing not only significantly enhances the genome coding capacity, but also serves as an efficient regulatory approach towards achieving developmental plasticity in plants [59]. Transcriptome is widely used to explore various biological mechanisms of plants, including leaf morphogenesis [34, 66]. In the present study, we conducted an in-depth analysis of transcriptome data at different developmental stages of leaves in *L. Chinense*. In order to explore the effects of transcriptional and post-transcriptional regulation on leaf morphogenesis, we compared differentially alternative splicing genes (DASGs) and differentially expressed genes (DEGs) among different leaf development stages. The results suggested that less overlap was observed between DASGs and DEGs (Fig. 4a-c). The enrichment analysis results also showed that DASG and DEG were rarely enriched in common pathways (Fig. 4f). According to previous studies, the post-transcriptional level and the transcriptional level may be independent regulatory mechanisms [6, 42, 67]. In fact, AS generates different mRNA splicing isoforms encoding protein products with different functions without affecting gene expression. Differentially expressed genes encode the same protein product but change its expression abundance. Therefore, both DASGs and DEGs may also play important roles in the establishment of leaf morphology. In addition, the protein products caused by AS may serve as regulatory signals to affect the expression patterns of other genes [68, 69]. Collectively, these findings indicate that the interaction between AS and transcriptional modulations is intricate and need further investigation.

Leaves originate from the shoot apical meristem (SAM) and undergo cells proliferation and expansion in three-dimensional polarity, ultimately forming its final size and shape [70]. Previous studies have shown that genes functioning at leaf initiation stage determine leaf morphology [71], which consistent with the results of GO enrichment analyses. DASGs and DEGs were significantly enriched in the GO terms associated with morphogenesis during the transition from fishhook (P2) to lobed (P7) leaves at leaf initiation stage, including ‘formation of plant organ boundary’, ‘formation of anatomical boundary’, ‘shoot system morphogenesis’ and more (Fig. 4f). Subsequently, in-depth analysis of the enriched genes revealed that the four DASGs related to leaf morphogenesis were derived from different transcription factor families, including BLH (BEL1-LIKE HOMEODOMAIN), ARF, (AUXIN RESPONSE FACTORS), and AIL/PLT (AINTEGUMENTA-LIKE/PLETHORA), which have been reported to be involved in leaf development or morphogenesis [27, 72, 73]. Besides, a comprehensive investigation of genes related to leaf morphogenesis revealed that some of them underwent AS events during leaf development involving various AS types, and eight of them identified as DASGs with difference between developmental stages (Fig. 6a, b, Supplementary Table S6). In summary, the findings indicated that key DASGs involved in leaf morphogenesis, weaving a complex regulatory network.

Our comprehensive analysis revealed that *AINTEGUMENTA-LIKE* (AIL) transcript factor, *LcAIL5*, undergoes AS regulation during leaf initiation, with a SE event of the fourth exon (Fig. 6c). AIL proteins belong to the APETALA 2/ETHYLENE RESPONSE FACTOR (AP2/ERF) family and contain two AP2 conserved domains. This variable exon, encoding the three amino acids residues ‘VYL’, is characteristic of microexons and is located in the first conserved AP2 domain of the AIL5 protein (Fig. 7b). Members of the AIL clade are commonly expressed in all dividing tissues of plants and play central roles in various developmental processes, including embryogenesis, stem cell niche specification, meristem maintenance, organ positioning and growth [74]. Prasad et al. investigated the function loss mutants of Arabidopsis *PLT3* (*AIL3*), *PLT5* (*AIL5*) and *PLT7* (*AIL7*) and discovered that these AIL proteins are key regulators of *PIN1* activity in controlling phyllotaxis [75]. Additionally, the *AIL5*/*PLT5* gene is expressed in the shoot tissues, including in vegetative and inflorescence shoot apical meristems (SAMs) [76]. These evidences indicate the important role of *AIL5* gene in early morphogenesis and meristem maintenance of young organs such as leaf and flowers.

Despite the confirmed function of *AIL5* in leaf morphogenesis in the model plant *A. thaliana*, the alternative splicing of this gene has not been explored. This study constructed a landscape of alternative splicing events in *L. chinense* through integrated transcriptome analysis during leaf shape development and discovered *AIL5* as a key candidate gene undergoing a SE event of 'VYL'. This is the first report of alternative splicing of *AIL5*. Additionally, analysis of AIL5 homologous proteins across the plant kingdom revealed similar alternative splicing events in other species such as *A. trichopoda* and poplar (*Populus trichocarpa* Torr. & Gray) (Fig. 7a). The ‘VYL’ motif is not only present in the AIL5 protein, but also in other AP2 proteins. Krizek et al. used a yeast system to test the ability of a randomly mutagenized population of Arabidopsis AINTEGUMENTA (ANT) to bind to ANT target sequences in yeast. Site-directed mutation of either “Y” or “L” residue leads to complete impairment of transcriptional activation, whereas mutation of the “V” residue results in reduced ANT activation [77]. This indicated that VYL plays an important role in binding to the target DNA sequence. Another AP2 family gene, *WRI1*, a key transcription factor in the regulation of plant oil synthesis, has the same exon skipping pattern in *Arabidopsis*, oil palm (*Elaeis guineensis* Jacq.), oat (*Avena sativa* L.) and castor (*Ricinus communis* L.). The study demonstrated that site-directed mutagenesis within the VYL motif in *AtWRI1* failed to restore the oil content in the *wri1* mutant, indicating that VYL is important for its function [78]. In this study, the VYL domain exhibited differential expression at different developmental stages. The ‘VYL’ inclusion isoform was upregulated during the P2 stage, while the ‘VYL’ exclusion isoform displayed highly expression at the P7 stage. This expression pattern suggests a potential phased and alternating involvement of these two transcripts in leaf morphogenesis. However, their specific functions require further experimental investigation.

## Conclusion

In summary, this study revealed the genome-wide alternative splicing (AS) landscape during leaf development in *L. Chinense* based on RNA-seq data and comprehensively deciphered the AS regulation mode of key genes involved in leaf morphogenesis. Based on the identified transcripts that are regulated by AS during leaf development, further functional characterization stands to enrich our understanding of the modulatory role of AS in leaf morphogenesis. Our findings also provide new perspectives and strategies for further deciphering the mechanism of plant leaf morphogenesis.

### Supplementary Information

**Additional file 1:** **Supplementary Table S1.** Mapping rates of all RNA-seq data.

**Additional file 2:** **Supplementary Table S2.** Types of splice junctions.

**Additional file 3:** **Supplementary Table S3.** Differentially alternative splicing genes (DASGs) and events during leaf development.

**Additional file 4:** **Supplementary Table S4.** Differentially expressed genes (DEGs) during leaf development.

**Additional file 5:** **Supplementary Table S5.** DASGs and DEGs enriched into morphogenetic related pathways.

**Additional file 6:** **Supplementary Table S6.** List of genes involved in leaf morphogenesis.

**Additional file 7: Supplementary Table S7.** The information about*AIL5* homologous genes from 58 plant species.
**Additional file 8:** **Supplementary File S1.** Updated annotation file in GFF3 format.
**Additional file 9:** **Supplementary File S2.** Genome wide alternative splicing events in *L. Chinense*.

## Data Availability

The Supplementary File S1 and File S2 are freely accessible through the Figshare database (10.6084/m9.figshare.25047311). The PacBio long reads and Illumina short reads were obtained from NCBI database with accession numbers of PRJNA559687, SRR8101043, SRR8101042, SRR8101041 and SRR8101040. All data generated or analyzed in this study are provided in this manuscript or supplementary information files.
